# Spatial summation of individual cones in human color vision

**DOI:** 10.1371/journal.pone.0211397

**Published:** 2019-07-25

**Authors:** Brian P. Schmidt, Alexandra E. Boehm, William S. Tuten, Austin Roorda

**Affiliations:** School of Optometry and Vision Science Graduate Group, University of California, Berkeley, CA, United States of America; University of Sussex, UNITED KINGDOM

## Abstract

The human retina contains three classes of cone photoreceptors each sensitive to different portions of the visual spectrum: long (L), medium (M) and short (S) wavelengths. Color information is computed by downstream neurons that compare relative activity across the three cone types. How cone signals are combined at a cellular scale has been more difficult to resolve. This is especially true near the fovea, where spectrally-opponent neurons in the parvocellular pathway draw excitatory input from a single cone and thus even the smallest stimulus projected through natural optics will engage multiple color-signaling neurons. We used an adaptive optics microstimulator to target individual and pairs of cones with light. Consistent with prior work, we found that color percepts elicited from individual cones were predicted by their spectral sensitivity, although there was considerable variability even between cones within the same spectral class. The appearance of spots targeted at two cones were predicted by an average of their individual activations. However, two cones of the same subclass elicited percepts that were systematically more saturated than predicted by an average. Together, these observations suggest both spectral opponency and prior experience influence the appearance of small spots.

## Introduction

A central goal of neuroscience is to understand how signals from sensory receptors are transformed into perceptual experience. In vision, photoreceptor cells in the retina encode real-time information about light in the environment. However, the signals conveyed by individual photoreceptors are noisy and ambiguous. A well-known example can be found in color vision. The spectral signals carried by individual cones are inherently ambiguous because each cone type is responsive to a relatively broad portion of the visible spectrum. As a result, a given magnitude of photoreceptor activity could result from virtually any combination of stimulus wavelength and intensity [1]. To extract color information from the photoreceptor mosaic, color-opponent neurons must compare the relative activity between cones with different spectral sensitivities [2–4]. Once a census of activity in the three cone types has been taken, the brain constructs a percept by inferring which stimulus most likely produced that activity pattern. In everyday viewing, our visual system navigates this process effortlessly, presumably by exploiting statistical regularities in the spatial, temporal and chromatic structure of natural images it has learned through experience.

The challenge of linking photoreceptor activity to object color can be laid bare in a laboratory setting by asking observers to judge the appearance of punctate stimuli that activate a small number of cones. Under these conditions, the color information carried by the cone mosaic is sufficiently restricted to induce cases where color perception is non-veridical. Krauskopf [5] reported dramatic fluctuations in the perceived hue of small, monochromatic flashes viewed foveally; due to apparatus limitations, ocular aberrations precluded the optical isolation of individual cones. Hofer et al. [6] found similar variability in the color sensations elicited by cone-sized, single-wavelength spots delivered through an adaptive optics system that corrected for ocular aberrations. While incessant fixational eye movements prevented direct knowledge of which retinal locus was being stimulated on each trial, the aggregate results nonetheless provided some clues about the strategies the brain may use to reconstruct spatiochromatic sensations from the trichromatic cone mosaic [7].

Recently, we have combined adaptive optics stimulation with high-speed retinal tracking to quantify the color appearance of cone-sized spots delivered repeatedly to cones of known spectral type [8–10]. A few general trends emerged from these studies. Firstly, the sensations elicited by targeting individual cones were repeatable across trials, suggesting that the variability observed in earlier studies arose from targeting variability caused by eye movements. Secondly, the spectral sensitivity of a probed cone was an important factor governing the elicited percept [9–11]. For example, against an achromatic background, reddish sensations were primarily associated with L cone stimulation, whereas greenish sensations more often resulted from targeting M cones. Together, these findings support the idea that the visual system can learn the spectral topography of the cone mosaic through accumulated experience [12].

A third striking outcome from these studies was that two cones with the same spectral sensitivity could elicit different sensations when probed with the same stimulus [6, 11]. Interestingly, Sabesan et al. [9] and Schmidt et al. [10] found clusters of cones that tended to evoke predominantly desaturated percepts. One interpretation of these results is that separate populations of cones feed into achromatic and chromatic pathways that are segregated in the retina [13, 14]. Under this scenario, the perception of, for example, a uniformly colored surface stimulating many cones would require the spread of color information from chromatic to achromatic regions, akin to a filling-in process. If a pair of neighboring cones—one “chromatic” and one “achromatic”—were stimulated together, one might predict the perceived hue would be governed solely by the color-signaling cone. An alternative explanation of the results of Sabesan et al. [9] is that each cone contributes to multiple post-receptoral pathways but with unequal weighting [15], such that a tendency for a particular cone to signal color might simply reflect a neural wiring bias that favors chromatic over achromatic circuits. In this scheme, the perceived hue elicited when the chromatic-achromatic pair are stimulated in tandem might instead resemble a weighted average of the sensations evoked when they are targeted singly.

Here, we examine these hypotheses by using a hue scaling paradigm to quantify color appearance when cones were stimulated individually or in pairs. First, increment threshold measurements were obtained to equate the detectability of the one- and two-cone stimuli. On average, detection mechanisms appeared to sum cone signals linearly, such that each cone received half the light dose in the paired stimulation condition compared to the single-cone case. With these stimuli, we found that the color appearance of small spots is influenced by the number and type of cones targeted. On average, when two L-cones were targeted they produced a slightly more saturated red percept than was predicted from the average of the sensations evoked by their individual activations. In comparison, an L- and M-cone activated together tended to elicit desaturated percepts. Together, these observations implicate a spectrally opponent mechanism that can be driven into a nonlinear regime when driven by high-contrast, single-cone stimuli.

## Methods

### Subjects

Three highly experienced subjects participated in the study. S10001 was a 34 year old male. S20075 was a 30 year old female. S20076 was a 31 year old male. All subjects had normal color vision (anomaloscope and Hardy-Rand-Rittler or Ishihara pseudoisochromatic plates) and were authors of the study. At the start of each session, cycloplegia and mydriasis were induced with drops of 1.0% tropicamide and 2.5% phenylephrine hydrochloride ophthalmic solution. Written consent was obtained from each subject before the experiments. All procedures were approved by the Institutional Review Board at the University of California Berkeley and adhered to the tenets of the Declaration of Helsinki.

### AOSLO microstimulator

A multi-wavelength adaptive optics scanning laser ophthalmoscope (AOSLO) was used to image and present stimuli to the retina. The AOSLO system [16–18] and the procedures for stimulating single cones have been described elsewhere in detail [9, 11, 19]. Briefly, light from a super-continuum laser (SuperK Extreme; NKT Photonics) was split into three channels with interference filters (Semrock): (1) A 940 nm channel was used to measure monochromatic aberrations. Light from this channel was collected into a wavefront sensor and that information was fed in real-time to a deformable mirror (DM97-08; ALPAO), which compensated for the measured aberrations. The resulting optical system was approximately diffraction-limited [16]. (2) An 840 nm channel was used to image the retina. Light from this channel was collected into a photo-multiplier tube (H7422-50; Hamamatsu) via a confocal pinhole and rendered into a video stream. (3) A 543 nm channel was used for retinally-targeted stimulation. L- and M-cones are approximately equally sensitive to this wavelength [20].

Retinal tracking was performed following the procedures of Arathorn et al. [21]. Briefly, the 840 nm video stream was registered to a reference image with a strip based cross-correlation procedure, which output retinal coordinates. Those coordinates were used to drive an acousto-optic modulator (Brimrose Corp.), a high-speed optical switch, which modulated the 543 nm channel. When the raster scan passed over a cell of interest the switch opened and delivered a calibrated dose of light to the cell.

Chromatic aberration between the three channels was measured and corrected with established procedures [22]. The imaging and stimulation rasters subtended a 0.95 degree field at a sampling resolution of ∼0.11 arcmin/pixel. The background in both experiments was white (CIE xy = 0.3, 0.32; 40 cd m^2^). Subject’s heads were stabilized with a custom-fit bite bar. For additional details on single cone stimulation and the accuracy of this procedure, Meadway and Sincich [23] recently published a detailed model of light propagation and capture by cone photoreceptors in AOSLO systems.

### Cone classification

In two subjects (S10001 and S20076), cones were classified according to their spectral type (L, M, S) using densitomety. The details of that procedure have been described elsewhere [8, 24]. The accuracy of these measurements is approximately 95% [8]. In one subject, we were unable to collect images with sufficient SNR to reliably classify cones.

### Day-to-day cone tracking

All experiments took place over multiple sessions and across multiple days. To return the same cones we used a three stage process. First, a high resolution image of the target retinal region was generated at the beginning of each session. The subject was asked to look toward a fixation cross positioned at a specific location relative to the AOSLO raster. Second, vascular or other coarse landmarks were used to identify the approximate location of the tested cells. Finally, a close-up visual inspection was used to find corresponding locations, cone-by-cone, between the current image and a reference image. Returning to the same cone is possible since, although the cone mosaic is generally close-packed, there are enough discontinuities in the packing for an unambiguous identification. The cone types, which are not identifiable in a grayscale AOSLO image, were labeled in the reference image for the two subjects that had been classified.

### Threshold measurements

Before quantifying appearance, we measured detection thresholds in the one and two cone conditions in order to control for differences in sensitivity. Detection thresholds (85% frequency of seeing) were measured with established procedures [19]. Experiments began by collecting a high SNR image from an average of 60-90 frames. Care was taken to select a region of the retina that would subsequently be used in appearance judgments. The experimenter then selected the center pixel of 8-12 contiguous cones from the reference image for testing. Thresholds were measured with an adaptive staircase procedure (QUEST) [25]. Each spot of light was monochromatic 543 nm, 0.35 arcmin (or 3x3 pixels) and was raster scanned against a low-photopic white background (40 cd/m^2^). In the case of paired stimulation, two spots of light, each 0.35x0.35 arcmin were delivered on each stimulus frame. Stimuli were presented over 500 ms (15 frames). The subject initiated each trial with a button press. An auditory beep indicated the start of the trial and then a stimulus was delivered to the center of either one or two of the selected cones. The subject reported whether she saw the flash with a single yes/no button press. No feedback was given. Each session consisted of four interleaved staircases. Two staircases measured single cone thresholds and two measured paired stimulation thresholds. Each staircase terminated after 35 trials. Stimulus order was randomized. On each trial one cone or one pair from the pre-selected group was targeted. Therefore, these measurements reflected an average threshold over the 8-12 cones. Thresholds for specific cones or pairs could not be estimated from this data, since each location was only targeted on a handful of trials. This approach was an efficient way to approximate thresholds over a larger group of cones and allowed us to proceed more quickly to appearance measurements, which were our primary interest.

A fraction of the 8-12 cones selected at the start of the experimental session were separated by multiple cones. Variable distances between cones in the selected region was a potentially confounding factor. To minimize its effect, threshold measurements were only made between pairs of cones separated by no more than one cone or roughly two arcmin between the center of each cone. Cones at this eccentricity are about 1 arcmin in diameter. At the end of each session, threshold energy was estimated from each staircase using the QUEST mean procedure [26]. This generated four threshold estimates: two for single cones and two for pairs. We then averaged thresholds within each condition and compared the threshold energy between two- and one-cone conditions. Subsequently, the threshold intensity measured for each subject was used in appearance experiments.

### Appearance judgments

Stimulus conditions in the appearance task were identical to the detection task. Flashes were 543 nm and 500 ms in duration and presented against a low photopic (40 cd m^2^), white background. The luminance of each spot was approximately 90 times higher than the background. Each spot of light was 0.35x0.35 arcmin and delivered approximately 6.04 log_10_ photons to the cornea. Experimental sessions began by capturing a high SNR image of the subject’s cone mosaic. From that image, three contiguous cones were selected for study. By selecting contiguous cones, we assured that cones were never separated by more than one cone (a center-to-center distance of ∼2 arcmin), which was the limit we set in the detection task. The subject initiated each trial with a button press, which was accompanied by an audible beep. On each trial, one or two of the selected cones were stimulated. The light energies used for one and two cone stimuli were set to each subjects’ previously determined detection thresholds. The recorded frequency of seeing in this task was 85.5%, as expected. Each cone and pair was tested 12 times for a total of 72 trials per session ([3 cones + 3 pairs] x 12 trials). Trials were randomly interleaved.

After each trial, subjects judged the hue and saturation with a scaling procedure [11, 27]. The subject indicated the percent of red, green, blue, yellow and white contained in each stimulus using five button presses such that each press represented 20% (5x20% = 100%). This response scheme is called five category scaling. One subject, S20075, used an alternative response schemed, called 4+1 category scaling [27]. In this procedure, the subject first rated saturation on a seven point scale. Then, hue was rated with five button presses using only red, green, blue and white. It has been shown previously that these two procedures produce very similar results, but some subjects prefer the 4+1 category approach [27]. Both results were converted into a common metric space as described below.

### Color appearance analyses

The raw color appearance dataset contained a total of 4,968 trials completed by three subjects. Before analyzing the data, unusable trials were removed. The location of the stimulus on each frame was recorded in real-time with a digital cross written into the video frames. To identify unusable trials, a delivery error was computed as the standard deviation of the stimulus location over the 15 frames (500 ms). Trials with delivery error greater than 0.35 or less than 0.01 arcmin (values below 0.01 do not occur naturally) were considered unusable. In those trials, we could not be confident that the correct cone was targeted. After removing bad trials (3.6%), 4,788 trials remained for further analysis. The remaining trials had a mean delivery error of 0.19 arcmin (standard deviation = 0.036 arcmin), which was about 1/5 of the diameter of cones at the eccentricities tested. Trials that either targeted an S-cone or were not detected were also removed. The remaining dataset contained trials in which individual or pairs of L- and M-cones were stimulated (N = 4,057). Finally, cones and pairs which had fewer than four good trials were not analyzed due to low statistical power. Most cones/pairs (71%) had at least 10 good trials.

Raw scaling data was transformed into a uniform appearance diagram [27]. For each trial, the number of red, green, blue, yellow and white button presses were converted to a percentage of the total button presses (five). A green-red dimension was computed as *gr* = (*green*% − *red*%)/100% and a yellow-blue dimension as *yb* = (*yellow*% − *blue*%)/100%. Saturation was computed from a sum of the absolute values of the green-red (gr) and yellow-blue (yb) dimensions (|*yb*| + |*gr*|). In 4+1 category scaling, each color category was scaled by the saturation judgment, which was normalized to range from 0-1. For example, consider a spot that was rated 60% red and 40% yellow at 40% saturation. Red and yellow, in this case, would be scaled down to 24% and 16%, respectively.

Analyses were carried out in the R programming language (https://www.r-project.org/).

## Results

The goal of these experiments was to determine how the visual system combines information across cones when making color judgments. To investigate this question, we probed L- and M-cones individually or in pairs with an AOSLO microstimulator. Before quantifying color appearance, we first measured detection thresholds in the one and two cone conditions and scaled our stimuli accordingly to ensure equal detectability across conditions. During appearance experiments, we used these measurements to set the stimulus energy level to achieve 85% frequency of seeing in both the one- and two-cone conditions.

### Detection thresholds sum linearly

Threshold energy (threshold intensity multiplied by stimulus area) for achieving 85% frequency of seeing (FoS) was determined with an adaptive staircase procedure. The values reported in Table 1 are the ratio of two:one cone threshold energies. This ratio equals one when the same energy (*i.e*. number of photons) was required to achieve threshold in both conditions. Values below one indicate less energy was necessary in the two cone case to achieve 85% FoS. The results from our three subjects were all close to one, which means, at threshold, each cone in a pair received approximately half the photons of the one cone case. Thus, the total energy was roughly equal across conditions and was consistent with linear summation. In subsequent experiments, individual and pairs of cones were stimulated at these threshold energies. Therefore, color judgments were made under conditions in which detection mechanisms were equally sensitive to all stimuli.

**Table 1:** Two:One cone threshold energy ratios.

subject	N	mean	StDv
S10001	4	0.98	0.09
S20075	4	1.05	0.12
S20076	11	0.92	0.1

N = the number of sessions completed. Each session contained two staircases for single cone stimulation and two for paired stimulation. After each session, the mean threshold energy in each condition was computed. The table reports the grand mean and standard deviation (StDv) of two:one cone ratios across all sessions.

### Variability in color perception

**Fig 1:**
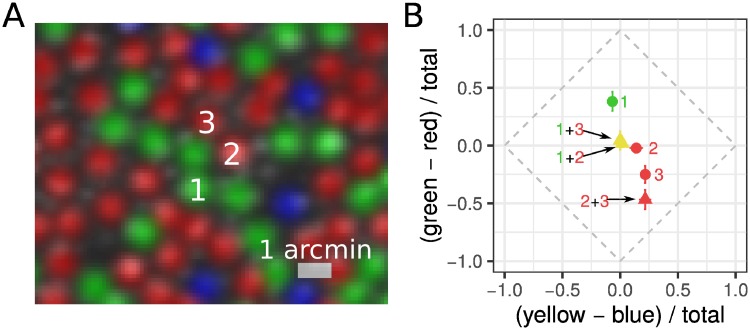
Measuring color appearance in one and two cone conditions. (A) Example AOSLO cone selection image (S20076; 1.5 degrees eccentricity). Groups of three cones were targeted during each experimental session (543 nm; 500 ms). Cones have been pseudo-colored to reflect their spectral type (red = L-cones, green = M-cones, blue = S-cones). The smaller, gray-scale blobs in between cones are rod photoreceptors. (B) Mean hue and saturation reports for one (circles) and two-cone (triangles) conditions. Numbers correspond to labels in (A). Results are plotted in a uniform appearance diagram (UAD), which represents bias towards the primary hues. An unbiased, or pure white, response falls at the origin. Green = M-cone(s), red = L-cone(s), yellow = L+M-cone pair. Error bars indicate ± SEM.

We next quantified color appearance of one and two cone spots presented at the measured threshold. Otherwise, stimuli were identical to those presented in the detection task. Previously, we have found no differences in brightness perception between L- and M-cones stimulated individually [11]. Three cones were selected for study in each session (Fig 1A). On each trial, either a single cone or a pair was targeted. After each flash, the subject judged the color of the spot using a hue and saturation scaling paradigm [11, 27]. Each cone and pair was tested twelve times. A total of 198 pairs were tested across three subjects. Hue and saturation scaling data were transformed into a color opponent representation. For each trial, the degree of perceived greenness versus redness and yellowness versus blueness was computed from percentage ratings as follows: *gr* = (*green*% − *red*%)/100% and *yb* = (*yellow*% − *blue*%)/100%. In this representation, saturation is expressed as the distance from the origin (in city block metric). A maximally saturated report falls along the outer diamond and a pure white response falls at the origin.

The results of one session are plotted in Fig 1B. In this example, Cone 1 was an M-cone and had a bias towards green (positive *gr* value). Cone 2 was an L-cone and elicited predominantly white reports. Cone 3, also an L-cone, was rated reddish-yellow (orange) with medium saturation (negative *gr* value, positive *yb* value). The percepts elicited when these cones were stimulated in tandem may provide insights into how the visual system combines color information across photoreceptors. In the example, when Cone 1 was targeted together with either Cone 2 or Cone 3, the average report had no clear color bias. In comparison, when Cone 2 and 3 were targeted they elicited a medium saturated orange report. Below, we analyze the results from all sessions and subjects.

**Fig 2:**
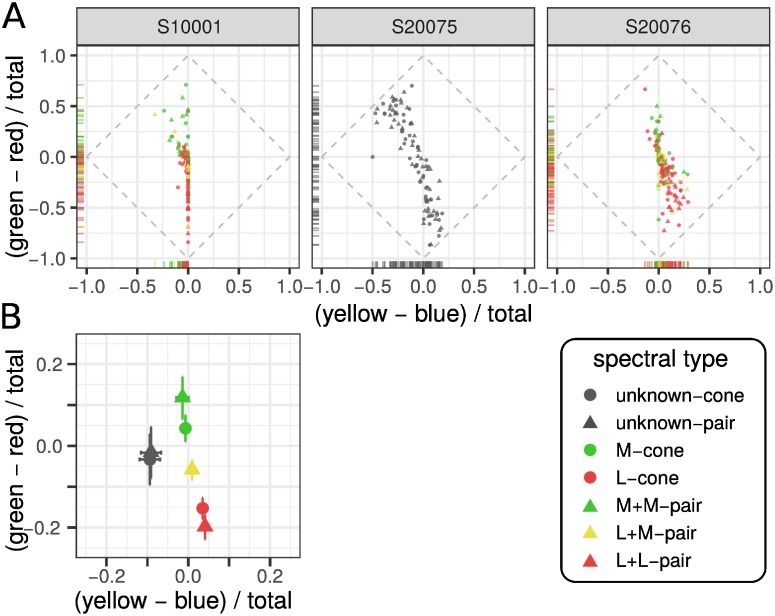
Number and type of cones probed influence color reports. (A) Average response from each cone and pair targeted in three subjects. Data was transformed into an opponent representation: yellow-blue and green-red. Marginal distributions are represented along each axis with rug plots. (B) The data from each subject was further grouped according to the cone type tested. The mean and standard error of each group are represented.

We first grouped each trial based on which cone or pair was probed. The results are reported in Fig 2A and separated by subject. Each point in these plots represents the mean response measured from a single cone or pair. This plot illustrates the variability in responses across cones/pairs and between subjects. There are a few features to note. Firstly, there were individual differences in color responses: S20075 used blue more frequently than the two other subjects and S10001 did not report yellow on any trials. However, the general patterns are similar. Most of the variance was found along the green-red dimension and there were few points that fell in the blueish-red or greenish-yellow quadrants. Secondly, in two subjects with classified mosaics, we found L-cone targeted trials were red biased, while M-cones were green biased. These patterns were similar to previous reports from single-cone [9–11] and large-field studies [28]. Thirdly, within a single subject, there was considerable variability between cones and pairs with the same spectral sensitivity. Similar variability in single cone mediated percepts has been reported previously [6, 9–11]. This is the first report of variability in percepts elicited from pairs of cones.

To better appreciate the influence of cone type and number of cones targeted on color reports, data was pooled across subjects and grouped according to the type of cone or pair probed. The mean and standard error for each group is shown in Fig 2B. When an individual or pair of M-cones was targeted the average *gr* response was greater than zero, indicating a bias towards green. In comparison, the average L-cone(s) elicited biases towards red and yellow. Together, these cone type specific differences in color reports were consistent with a predictive relationship between cone type and color report, as previously reported [9, 11]. Two cones with the same photopigment tended to elicit slightly more saturated reports than single cone trials. On the other hand, one L- and one M-cone targeted together tended to produce desaturated reports.

### Mosaic parameters do not predict percepts

Fig 2A illustrates that color reports varied even between cones with the same photopigment. Some L-cones, for instance, elicited saturated red percepts, while a majority produced white or desaturated red reports. We next asked whether this variability could be explained by features of the mosaic. Specifically, can we predict whether an L-cone will produce a saturated or a desaturated red based on the surrounding cone types? And in the case of paired stimulation, did the distance between the two cones influence color appearance? The existence of such relationships could implicate low-level neural mechanisms, such as chromatically-opponent ganglion cells, in this behavior.

The local neighborhood surrounding a cone is thought to be an important factor influencing color percepts associated with small spots [29]. To test this prediction, we found the number of L-cones in the immediate neighborhood of each cone/pair. In keeping with prior work [9–11, 30] the local neighborhood was defined as the six nearest cones. In the case of a pair, the immediate neighborhood for each cone was found separately and duplicates were removed. We did not find a significant correlation between the number of neighboring L-cones and the mean response in any dimension (*gr*, *yb* or saturation)(*p* > 0.05).

The distance separating two cones in a pair may also be an important factor influencing appearance. However, this measure was not correlated with hue or saturation reports (*p* > 0.05). Cone pairs were never separated by more than one cone, which may explain why we did not detect a relationship. Moreover, subjects verbally reported that the flashes always appeared as a single uniformly colored dot. In the future, systematically varying the distance between stimulated pairs will be an informative exercise. At a certain critical distance, the spots of light will be seen as two spatially distinct dots. It is less clear at what distance the spots will be perceived as two distinct colors.

### Paired simulation was predicted by an average of individual reports

We next sought to address how color signals were combined in the two cone condition. As elaborated in the Introduction, there were two broad hypotheses: 1) Separate populations of cones feed into color and achromatic circuits. When a “color” and “achromatic” cone are stimulated together the resulting percept should be determined by the “color” signaling cone alone. 2) Each cone may contribute to both color and achromatic pathways with different weights. In this case, two cone color reports should be predicted by an average of the percepts elicited from their individual activations.

**Fig 3:**
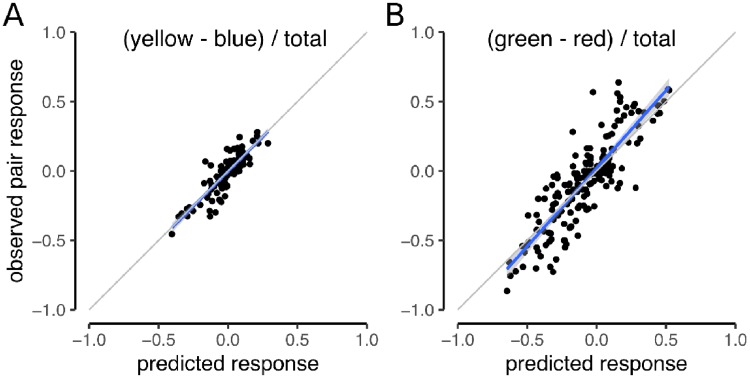
An average of individual responses predicts paired stimulation. The response measured for each pair was predicted from the average response of the two cones tested individually. Blue line represents the best fit line with 95% confidence intervals indicated by gray shading. (A) Blue-yellow (*by*) dimension. Best-fit line: *yb*_*observed*_ = 0.004 + 1.002*yb*_*predicted*_. (B) Green-red (*gr*) dimension. Best-fit line: *gr*_*observed*_ = 0.02+ 1.12*gr*_*predicted*_. Gray lines indicate unity slope. Analyses include data from all three subjects.

To distinguish between these two hypotheses, we matched the mean response from each cone pair with the mean report from each cone tested individually. We then fit an averaging model to the data. Behavioral reports, *r*, from two-cone stimulation were predicted by an average of the individual responses: *r*_12_ = (*r*_1_ + *r*_2_)/2. Predictions were computed for *gr* and *yb* dimensions separately. Fig 3 shows the measured responses plotted against these predictions. An average of the single cone responses was a good fit to the data in both the *gr* (R^2^ = 0.73; *p* < 0.01) and *yb* (R^2^ = 0.75; *p* < 0.01) dimensions. The best fit lines had slopes close to unity, which further supported the hypothesis that an average of individual responses was a good model.

### Cone-pairs with the same photopigment elicit saturated percepts

**Fig 4:**
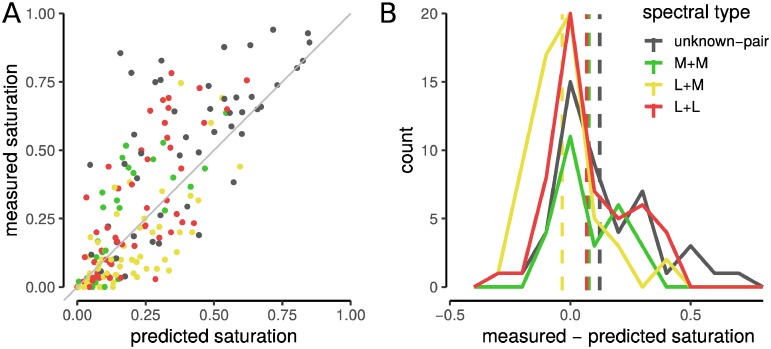
Cone pairs with the same spectral sensitivity produce higher saturation ratings than predicted. Saturation judgments were predicted for each measured cone pair with a linear average model. (A) Model predictions were plotted against the mean saturation ratings measured for each pair. Gray line indicates a prediction that matches the measured judgment exactly. (B) Distribution of measured saturation judgments minus predicted responses. Dotted lines indicate the mean of each distribution. Colors indicate cone type of pair: red = L+L, green = M+M, yellow = L+M, gray = unknown.

While an averaging model captured a large fraction of the variance in two-cone color judgments, there were some pairs that deviated substantially from the best fit line. We wondered whether these deviations from an average might be predicted by the sub-class of the two cones. For instance, were L+M-pairs more likely to deviate from the model? To answer this question, we found the saturation for each pair and compared it to the saturation predicted by the average of the two cones probed alone (Fig 4A). A unity line represents the condition where the observed saturation judgment was predicted exactly by an average of individual responses. Notice that the L+L and M+M pairs tended to lie above the unity line, particularly at higher saturation values. In contrast, the L+M pairs often fell below the line. These observations indicate that cones of the same spectral type produced slightly more saturated reports than predicted by the average of their individual responses.

We quantified this trend directly by taking the difference between the observed and predicted saturation judgments. The results are illustrated in a histogram (Fig 4B). Two-tailed t-tests confirm that the L+L and M+M pairs were significantly more saturated than an average of their individual responses (mean = 0.072, *t*_78_ = 4.2, *p* < 0.01). In comparison, the mean difference for L+M pairs approached significance in the opposite direction (mean = -0.034, *t*_56_ = -1.9, *p* = 0.06). These pairs were slightly more likely to be less saturated than predicted by an average of the individual responses. Across all of the unclassified cones tested in S20075, the average pair was more saturated than an averaging model predicted (mean = 0.123, *t*_46_ = 4.0, *p* < 0.01). It is worth noting that this dataset contains all combinations of L and M cones. Had L+M pairs been removed from the S20075 dataset, the difference between observed and predicted saturation judgments may have been even more pronounced.

## Discussion

We quantified the color appearance of small spots of light targeted to individual or pairs of cones. Our experiments revealed that both the number and spectral type of targeted cones influenced color reports (Fig 2). Generally, pairs of cones elicited colored percepts that were predicted by an average of individual responses (Fig 3). This finding suggests that each cone contributes to the post-receptoral circuits involved in color vision and is inconsistent with the view that a subgroup of cones are the sole stakeholders in the processes responsible for generating hue sensations.

Targeted in isolation, we observed that individual L and M cones often produced reddish and greenish sensations, respectively (Fig 2). This result was consistent with previous work in which the color appearance of small spots was studied [6, 9, 11]. When opposite-type cone pairs (i.e. L+M) were stimulated together the evoked percept was, on average, desaturated, or white (Fig 4). Mixtures of red and green were never perceived by our subjects. These findings uphold a fundamental tenet of classical opponent-process color theory [31, 32] and implicate a role for spectrally-opponent mechanisms in color perception at the cellular scale [33].

Where in the visual pathway might cone signals from different spectral classes be pitted against each other? One candidate site is in the outer retina, where horizontal cells mediate lateral inhibition between nearby cones [34, 35]. Spatial antagonism in the outer retina could contribute to two-cone stimuli appearing less saturated than single-cone spots if the pair of engaged cones inhibited each other mutually, thereby reducing the magnitude of their respective outputs. Because horizontal cells receive non-selective inputs from L and M cones [36], any relative desaturation of two-cone stimuli mediated by horizontal cells should be observed in both opposite- and like-type cone pairs. In contrast, we found that homologous (e.g. L+L) cone pairs tended to produce sensations that were more saturated than the predictions generated by the simple averaging model (Fig 4), thus arguing against the outer retina as the site of the non-linear summation we observed.

Instead, our results suggest the critical comparison between cone types takes place downstream from the first visual synapse. The red-green dimension of color perception is thought to depend on signals originating in midget retinal ganglion cells. At the eccentricities examined here, midget RGCs draw excitatory input from individual L and M cones and feature concentric receptive field surrounds. This private-line, center-surround wiring scheme leaves midget cells responsive to both achromatic and chromatic modulations, albeit with different spatial tuning [37]. Color and luminance information can be decoded from multiplexed midget signals by additional processing at a post-retinal stage. Various demultiplexing frameworks have been proposed (for a review, see [38]), but their general form involves separate pathways that sum and difference L-On and M-On (or L-Off and M-Off) mechanisms to extract achromatic and chromatic signals, respectively. The differencing operation could account for how the color information carried by individual L and M cones is lost when they are stimulated together. The summative mechanism, along with other visual pathways that pool signals from a larger number of cones (e.g. magnocellular neurons), could explain why detection performance for one- and two-cone stimuli exhibited linear summation (Table 1).

A second interesting question raised by the results of the present study is why L+M paired stimulus did not elicit strong sensations along the blue-yellow dimension of color vision. In the standard three-dimensional color space defined by one non-opponent (L+M) and two color-opponent (L-M and S-(L+M)) axes, stimuli that increase the cumulative activity in L and M cones reside in a plane of higher luminance [39, 40]. If L and M cone activity is elevated equally relative to an achromatic background, such that the difference in their activations remains constant, the higher-luminance stimulus is restricted to lie along a S-(L+M) axis that spans bluish to yellowish colors, with an achromatic point in between. The location of the stimulus along this axis depends on the relative level of S cone activity. Under ordinary viewing conditions, optical aberrations, natural image statistics, and fixational eye motion combine to ensure that most stimuli will be sampled by at least one S cone, thus providing the brain with a reliable short-wavelength signal that can be used to assign the color appearance. In contrast, the two-cone stimulus used in this study precludes a direct assay of S cone activity, forcing the visual system to infer stimulus color using signals from just two cone types. Our data suggest that under these conditions, the brain tends to interpret an elevation in L and M cone activity as an achromatic luminance modulation. This result is consistent with an earlier study in which small, middle-wavelength adaptive optics flashes presented against a dark background were often seen as achromatic and only occasionally judged to be yellow [6].

The most surprising finding of the present work was that when two cones of the same type were probed, subjects reported seeing a hue that was more saturated than an average of the two probed alone (Fig 4). This was unexpected because the stimuli were adjusted to be equally detectable (Table 1). Thus, while detection performance was equated across stimulus conditions, the color percept was influenced by activity in a second cone and saturation was systematically elevated. One possible mechanistic explanation for the increased saturation we observed in two cone stimulation (Fig 4) is the presence of a saturating non-linearity before cone signals are summed. Horwitz and Hass [41] described color cells in primary visual cortex that compressed cone inputs before summation in a manner consistent with our observations. In comparison, our threshold measurements followed a linear summation model, which is consistent with the area of complete summation (Ricco’s area) at this eccentricity [42]. Together, our observations support the idea that separate neural mechanisms mediated these two tasks [43].

The approach used here of targeting small groups of cones provides a means of testing sophisticated hypotheses about neural mechanisms and their role in shaping visual experience. Our evidence supports the idea that the appearance of small spots is dependent upon both the number and type of cones targeted. These observations are consistent with different strategies for combining information within versus across neuronal sub-classes. In the future, scaling these experiments to larger groups of cones will provide important clues about how the visual system extracts color and spatial signals in more naturalistic settings.
